# Up in Arms: Immune and Nervous System Response to Sea Star Wasting Disease

**DOI:** 10.1371/journal.pone.0133053

**Published:** 2015-07-15

**Authors:** Lauren E. Fuess, Morgan E. Eisenlord, Collin J. Closek, Allison M. Tracy, Ruth Mauntz, Sarah Gignoux-Wolfsohn, Monica M. Moritsch, Reyn Yoshioka, Colleen A. Burge, C. Drew Harvell, Carolyn S. Friedman, Ian Hewson, Paul K. Hershberger, Steven B. Roberts

**Affiliations:** 1 Department of Biology, University of Texas at Arlington, Arlington, Texas, United States of America; 2 Department of Ecology & Evolutionary Biology, Cornell University, Ithaca, New York, United States of America; 3 Department of Biology, Pennsylvania State University, University Park, Pennsylvania, United States of America; 4 Donald P. Shiley Bioscience Center, San Diego, California, United States of America; 5 Marine Science Center, Northeastern University, Nahant, Massachusetts, United States of America; 6 Department of Ecology and Evolutionary Biology, University of California, Santa Cruz, California, United States of America; 7 School of Aquatic & Fishery Sciences, University of Washington, Seattle, Washington, United States of America; 8 Department of Microbiology, Cornell University, Ithaca, New York, United States of America; 9 U. S. Geological Survey, Western Fisheries Research Center, Marrowstone Marine Field Station, Nordland, Washington, United States of America; Chang Gung University, TAIWAN

## Abstract

Echinoderms, positioned taxonomically at the base of deuterostomes, provide an important system for the study of the evolution of the immune system. However, there is little known about the cellular components and genes associated with echinoderm immunity. The 2013–2014 sea star wasting disease outbreak is an emergent, rapidly spreading disease, which has led to large population declines of asteroids in the North American Pacific. While evidence suggests that the signs of this disease, twisting arms and lesions, may be attributed to a viral infection, the host response to infection is still poorly understood. In order to examine transcriptional responses of the sea star *Pycnopodia helianthoides* to sea star wasting disease, we injected a viral sized fraction (0.2 μm) homogenate prepared from symptomatic *P*. *helianthoides* into apparently healthy stars. Nine days following injection, when all stars were displaying signs of the disease, specimens were sacrificed and coelomocytes were extracted for RNA-seq analyses. A number of immune genes, including those involved in Toll signaling pathways, complement cascade, melanization response, and arachidonic acid metabolism, were differentially expressed. Furthermore, genes involved in nervous system processes and tissue remodeling were also differentially expressed, pointing to transcriptional changes underlying the signs of sea star wasting disease. The genomic resources presented here not only increase understanding of host response to sea star wasting disease, but also provide greater insight into the mechanisms underlying immune function in echinoderms.

## Introduction

Echinoderms provide a powerful system for examining invertebrate immune function from both an organismal and evolutionary perspective. With a taxonomic position at the base of the deuterostomes, echinoderms are more similar to chordates and vertebrates than to other invertebrate lineages [1]. Echinoderms possess a complex immune system with specialized phagocytic cells, signaling molecules that circulate in coelomic fluid, and a melanization response [2–4]. The eponymous cells within the coelomic fluid, the coelomocytes, are critical for both cellular and humoral elements of the immune response, including phagocytosis and the release of antimicrobial enzymes to target and destroy foreign antigens [4]. Laboratory studies have established the existence of echinoderm recognition proteins [5, 6], cytokine signaling molecules [7], and effector mechanisms, such as antimicrobial peptides [8]. Conserved immune response pathways identified in genomic and proteomic screens of echinoderms include the complement system, Toll pathway, and cell adhesion regulation [3, 9, 10]. While the discovery of these major pathways highlights broad similarities across the phylogenetic tree, echinoderms also have regions of unusually high diversification in some innate immune genes. For example, unlike *Drosophila melanogaster*, which possesses 9 Toll-Like Receptor (TLR) genes, the sea urchin *Strongylocentrotus purpuratus* possesses over two hundred TLR genes [9]. Other genes suggest overlap with vertebrate immunity, such as homologs of RAG1 and RAG2 genes, which are involved in diversifying recognition capabilities of vertebrate lymphocytes and are important in response to pathogens and disease response [9].

Infectious diseases of marine echinoderms are on the rise [11], providing an opportunity to investigate the varied immune responses of these animals to multiple pathogens. This rise in epizootics further underscores the need for a thorough understanding of the echinoderm immune system. Sea star wasting disease (SSWD) has previously caused mass mortalities in over 12 species of asteroids in southern California-Baja California and in the Puget Sound-Vancouver Island area [12, 13]. The 2013–2014 SSWD outbreak, with a broader host (20 species) and latitudinal range than previously observed [14], has caused severe population declines in multiple sea star species along the North American Pacific coast [15]. Early signs of SSWD, referred to here as a “wasting phenotype”, include abnormally twisted arms, a deflated appearance, and white lesions on the aboral body wall; this rapidly progresses to tissue degradation, structure loss, arm loss, and death [12]. New evidence implicates a denso-virus as the causative agent in the 2013–2014 outbreak [14]. Many basic details of SSWD are still unknown, including the mode of pathogen spread, host pathogen interactions, and conditions influencing its severity [15]. Two of the most affected species, *Pisaster ochraceus* and *Pycnopodia helianthoides* are considered to be keystone species [16, 17]; loss of keystone asteroid populations to disease outbreaks has the potential to shift community composition of intertidal and subtidal ecosystems [16, 17]. Removing *P*. *ochraceus* predation can lead to community dominance of their preferred prey species, the California mussel, *Mytilus californianus* [18]. Without *P*. *helianthoides* to break up urchin aggregations, urchins can overgraze kelps, transforming healthy kelp forests into urchin barrens [17, 19].

In this study, we characterize the sea star immune response in the sunflower star, *Pycnopodia helianthoides*, following injection of asymptomatic individuals with homogenized, virus size-fractionated (<0.2 μm) filtrates from sea stars with signs of wasting disease. The *P*. *helianthoides* transcriptome generated here is the first immune-related transcriptome in the class Asteroidea. This transcriptome not only provides an important resource for future evolutionary and organismal studies, but also provides the first evidence of how sea stars mount a response to this devastating disease. Understanding the host response to this rapidly progressing disease will provide insight into dynamics of host-pathogen interactions in marine systems and lay the foundation for future conservation efforts. Furthermore, this exploration of the sea star response to disease can enhance our understanding of the evolution of the immune response.

## Materials and Methods

### Experimental Design

*Pycnopodia helianthoides* (radius 160.5 +/- 30.9 mm) were collected from four sites in Washington State: South Whidbey Harbor at Langley (LA) (48.038, -122.404); Dabob Bay (DB) (47.813, -122.820); Port Hadlock Marina (PH) (48.030, -122.745); Friday Harbor Laboratories (FH) (48.545, -123.012). The Washington Department of Fish and Wildlife issued collection and transfer permits to the University of Washington's Friday Harbor Lab for the collection of the sea stars used in this study. Collections on the San Juan Islands were conducted with permission on Friday Harbor Lab property. Collections at Dabob Bay were conducted on tidelands owned by Taylor Resources with their permission. Collections at South Whidbey Harbor at Langley and Port Hadlock Marina were conducted with permission from each site’s respective harbormaster. *P*. *helianthoides* is not a protected or endangered species. Sites had no reports of SSWD infection at time of collection. An effort was made to collect animals within a similar size range to reduce variation based on body mass but otherwise selection was random. Experiments were conducted at the US Geological Survey, Marrowstone Marine Field Station. *P*. *helianthoides* were transported to the lab on the day of collection in coolers, wrapped in cloth soaked in seawater from their respective sites.

Upon arrival, animals were examined for signs of disease or trauma and then transferred to individual, 37.8 L aquariums with separate flow-through, sand-filtered, and UV-treated seawater at 30–31 psu and maintained at ~9.5°C. The acclimation period for each star varied based on collection date: 3 days (Friday Harbor); 1 month (Port Hadlock marina) 2.5 months (Langley, Dabob Bay), depending on time of collection. During this period, animals were examined for signs of disease and fed live manila clams (*Ruditapes philippinarum*), every 3–4 days.

Individuals were inoculated with tissue homogenate prepared from the tube feet, dermal tissue, and coelomic fluid of three *P*. *helianthoides* with clinical signs of SSWD. The tissue was ground with a mortar and pestle, homogenized in a Stomacher with 10 mL seawater, and centrifuged at 1000 x *g* for 5 min at 4°C. The resulting supernatant was divided into two aliquots: treatment and control (in the latter any viable cells were heat-killed by boiling for 7 mins prior to use). Both homogenates were filtered to a viral-sized fraction (0.22 um polyethylfulfone filter), and organisms were injected with either the control or treatment viral-sized fractions. Injections were made into the coelomic cavity, under the dermis on the aboral side of the arm, and at the arm base. Animals were checked twice daily and any physical or behavioral changes recorded. All animals were sacrificed 9 days post-injection once all three treatment animals showed small lesions, the first visible signs of SSWD. This sampling point was determined based upon symptom observation, so that we knew animals were infected, however it does preclude our ability to detect early host response activity and delineate general stress responses from virus specific responses. Stars were sampled while lesions were small and they still had turgor so the coelomic fluid could be extracted from an intact animal. Coelomic fluid was collected from the interradius between the inferomarginal and superomarginal ossicles (the “arm pit”) using a syringe. The samples were centrifuged for 5 minutes at 1200 rpm at 4°C to separate the coelomocytes. The fluid was then removed and the pellet containing the coelomocytes flash frozen in liquid nitrogen and stored at -80°C.

### High Throughput Sequencing and Assembly

Total RNA was extracted using Tri-Reagent per manufacturer’s instruction. Potential DNA carryover was reduced using the Turbo DNA-free treatment according to the manufacturer's instructions (Ambion). RNA quality, library preparation, and sequencing were performed by the Cornell University Institute for Biotechnology. RNA quality was assessed using an Advanced Analytical Fragment Analyzer. Libraries were prepared using the Illumina TruSeq RNA Sample Preparation kit according to the manufacturer’s protocol (including bar-coding for multiplexing), except our sample concentration was between 99–1503 ng per sample. Samples were multiplexed where six samples were run in one lane for Illumina HiSeq 100 bp paired end sequencing.

Quality trimming of resulting sequencing reads was performed using CLC Genomics Workbench v. 7.0 (CLC Bio, Germany) with the following parameters: quality limit = 0.05, number of ambiguous nucleotides < 2 on ends, reads shorter than 20 bp were removed, and Illumina PCR primers were removed. *De novo* assembly was performed with Genomics Workbench 7.0 (CLC Bio, Germany) on quality trimmed sequences with the following parameters: automatic bubble size, automatic word size, auto-detect paired distances, perform scaffolding, and minimum contig size of 500 bp. To remove any bacterial sequences from the transcriptome data, consensus sequences were compared to the NCBI nt database using the BLASTn algorithm. Consensus sequences with significant matches (e-value = 0) to bacterial sequences were removed (n = 1102) and not considered in subsequent analyses.

### Transcriptome Characterization

In order to characterize relative completeness of the transcriptome, it was compared to complete transcriptomes available for the bat star *Patiria miniata* (http://echinobase.org) and purple sea urchin *Strongylocentrotus purpuratus* (http://spbase.org) [20]. Specifically, gene sets of *P*. *minata* (29,805) and *S*. *purpuratus* (31,159) were used as the query for BLASTn comparisons to our *P*. *helianthoides* transcriptome. The *P*.*miniata* fasta file was downloaded in August 2014 as Pm_genes.fasta.zip from Echinobase and is available in our accompanying repository [21]. The *S*. *purpuratus* fasta file was downloaded in August 2014 as SPU_Nucleotides.fasta from SpBase, and is in our accompanying repository [21].

*P*. *helianthoides* transcriptomic sequences were annotated by comparing contiguous sequences to the UniProtKB/Swiss-Prot database. Comparisons were made using the BLASTx algorithm with a 1.0E-5 e-value threshold. Genes were classified according to Swiss-Prot Gene Ontology (GO) associations, as well as respective parent categories (GO Slim). Annotation analyses and data are published in accompanying GitHub repository [21].

### Differential Expression Analysis

Read counts were determined by aligning reads with CLC Genomics Workbench v. 7.0 with the following parameters: mismatch cost = 2, insertion cost = 3, deletion cost = 3, length fraction = 0.8, similarity fraction = 0.8, maximum number of hits for a read = 10. Differential expression of contigs was calculated using a negative binomial GLM in the R package DESeq2 [22]. The read counts were first normalized using the size factors method and fit to a negative binomial distribution. Significantly differential contig expression (Benjamini-Hochberg adjusted p<0.05) between control and treated animals was determined using the Wald test for significance of GLM terms. Count data and code used for differential expression analysis are published in accompanying GitHub repository [21]. Heatmaps were generated from the normalized read counts produced by DESeq2 using the program GENE-E (http://www.broadinstitute.org/cancer/software/GENE-E/) and clustered by pair-wise distance.

### Enrichment Analysis

Enriched GO terms associated with differentially expressed genes were identified using the Database for Annotation, Visualization and Integrated Discovery (DAVID) v. 6.7 [23]. The specific GO category (GO FAT), developed as part of the Annotation Tool of the DAVID suite of bioinformatics resources, is a category that filters out very broad GO terms based on a measured specificity of each term. Analysis was performed on annotations associated with the Biological Process domain. Specifically, UniProt accession numbers for differentially expressed genes were uploaded as a gene list, while UniProt accession numbers for all annotated contigs were used as a background. Enriched GO terms were identified as those with Benjamini-Hochberg adjusted p<0.05.

## Results

### Inoculation Experiment

During the acclimation period, no signs of disease were observed. All treated *P*. *helianthoides* developed signs of SSWD including curling and lesions. Lesions were noted at 8 to 9 days post injection in all three treated animals. Although individuals varied slightly in the timing of onset and duration, clinical signs were consistent among animals [14]. Control animals did not show any clinical signs.

### Transcriptome

This sequencing effort resulted in a combined 2.9x10^8^ paired end reads among all six libraries (three control individuals and three treated individuals). Sequencing reads are available in NCBI SRA Accession # SRP051104. After quality trimming, reads were assembled into 29476 consensus sequences with an N50 value of 1757 bp. In order to assess how much of the full gene repertoire was sequenced, the *P*. *helianthoides* transcriptome was compared to both the asteroid *Patria miniata* and the echinoid *Stronglycentrotus purpuratus* complete transcriptomes. We found that 52% and 26% of the respective transcriptomes matched the *Pycnopodia helianthoides* transcriptome using a 1.0E-5 e-value threshold. Comparison of the *P*. *helianthoides* transcriptome to the UniProtKB/Swiss-Prot database resulted in annotation of 10513 contigs. **S1 Table** provides UniProtKB/Swiss-Prot and Gene Ontology annotation information. In addition, annotation analyses and corresponding files are published in accompanying GitHub repository [21].

### Differentially Expressed Genes

**Fig 1:**
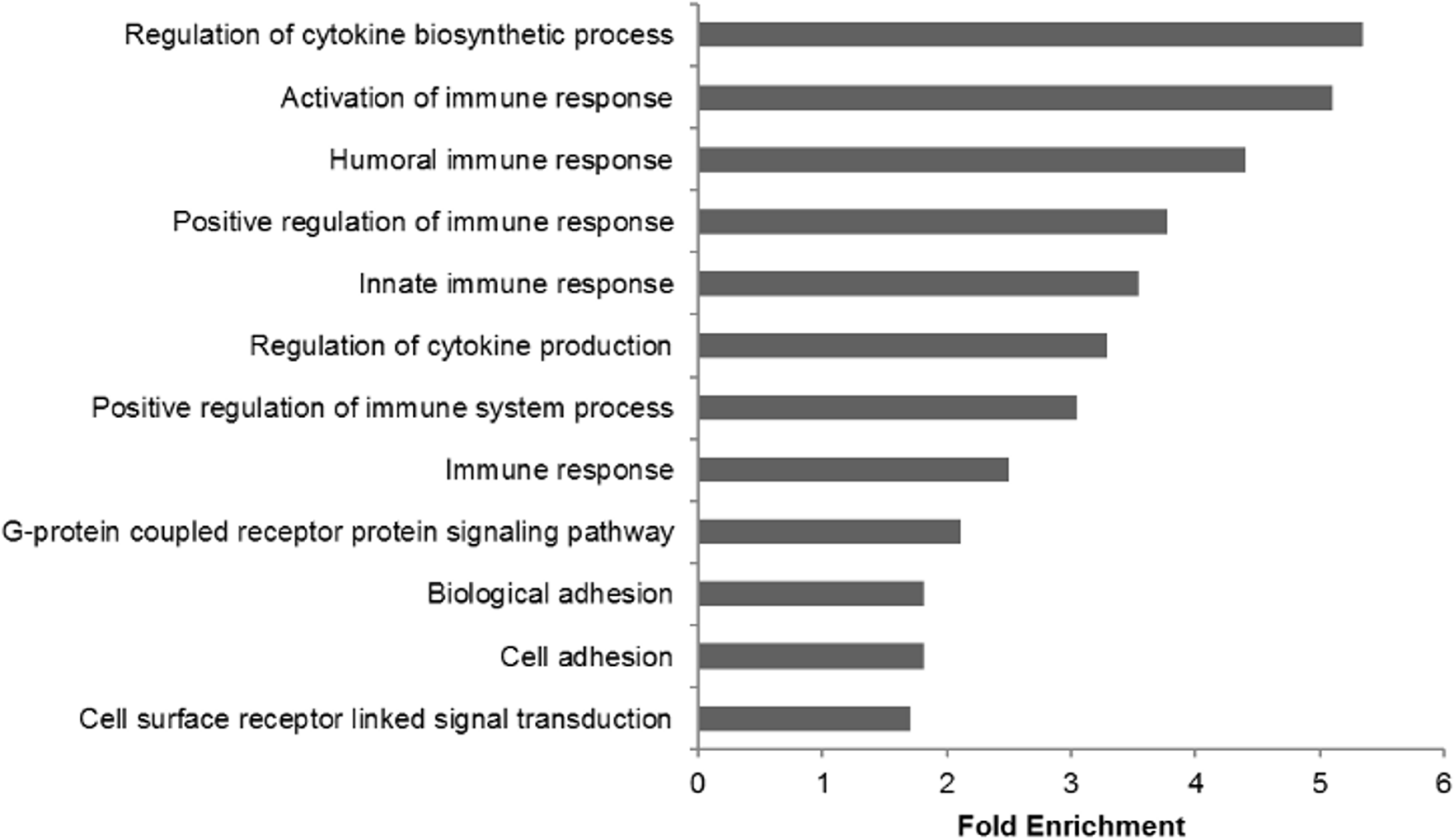
Differential gene expression between treated and control *P*. *helianthoides*. Fold change (Log_2_) expression between treated and control organisms is plotted where positive values represent contigs expressed at higher level in treated sea stars. Red circles indicate those contigs determined to be differentially expressed (padj < .05; n = 3773). Differential expression of contigs was calculated using a negative binomial GLM in the R package DESeq2.

Of those contigs identified as differentially expressed (n = 3773), in treated individuals, 1,629 were expressed at lower levels and 2,103 were expressed at higher levels than in control individuals (**Fig 1**). A total of 1183 differentially expressed contigs (31.7%) were annotated based on comparison to the Uniprot/SwissProt database.

**Fig 2:**
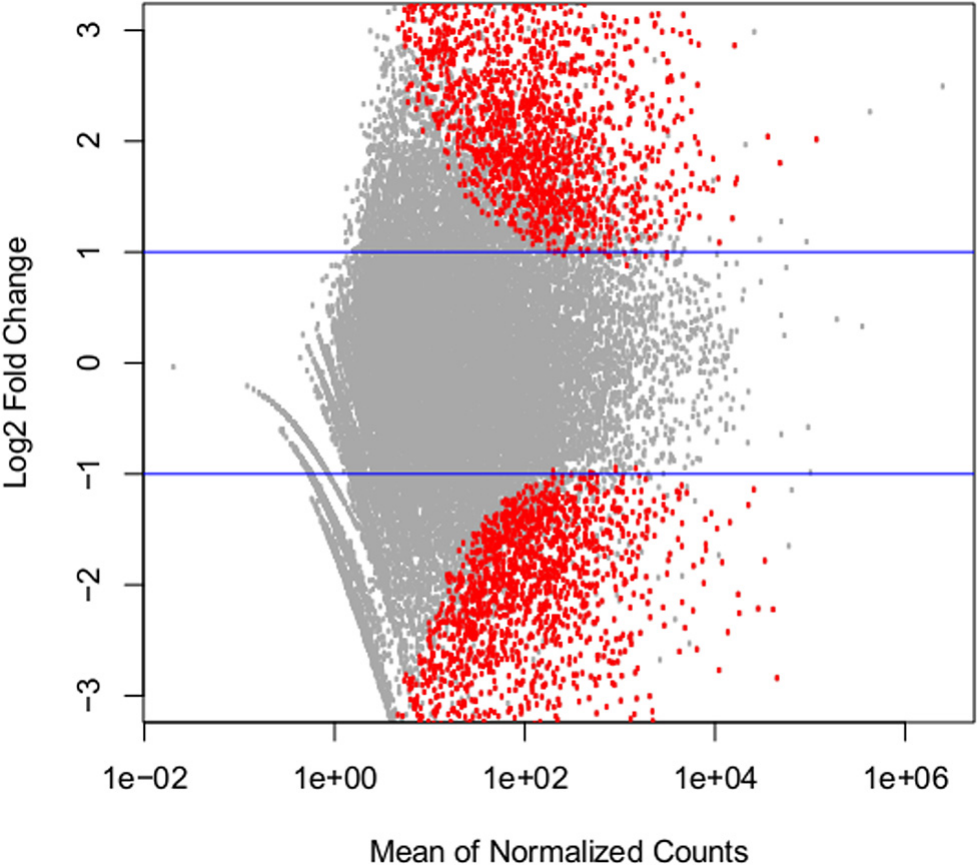
Fold enrichment of significant gene ontology terms. Fold enrichment of significantly enriched (padj < .05) biological process GO terms.

Seventeen gene ontology terms were significantly enriched based on the subset of annotated differentially expressed genes. These terms fell into three broad categories: immune response, regulation of cytokine production, and biological adhesion. Immune response was the largest of these categories, with thirteen associated enriched GO terms. **S1 Table** lists all significantly enriched GO terms. Regulation of cytokine biosynthetic process (GO:0042035) and activation of immune response (GO:0002253) were the two most enriched GO terms with fold enrichments >5 (**Fig 2**).

**Fig 3:**
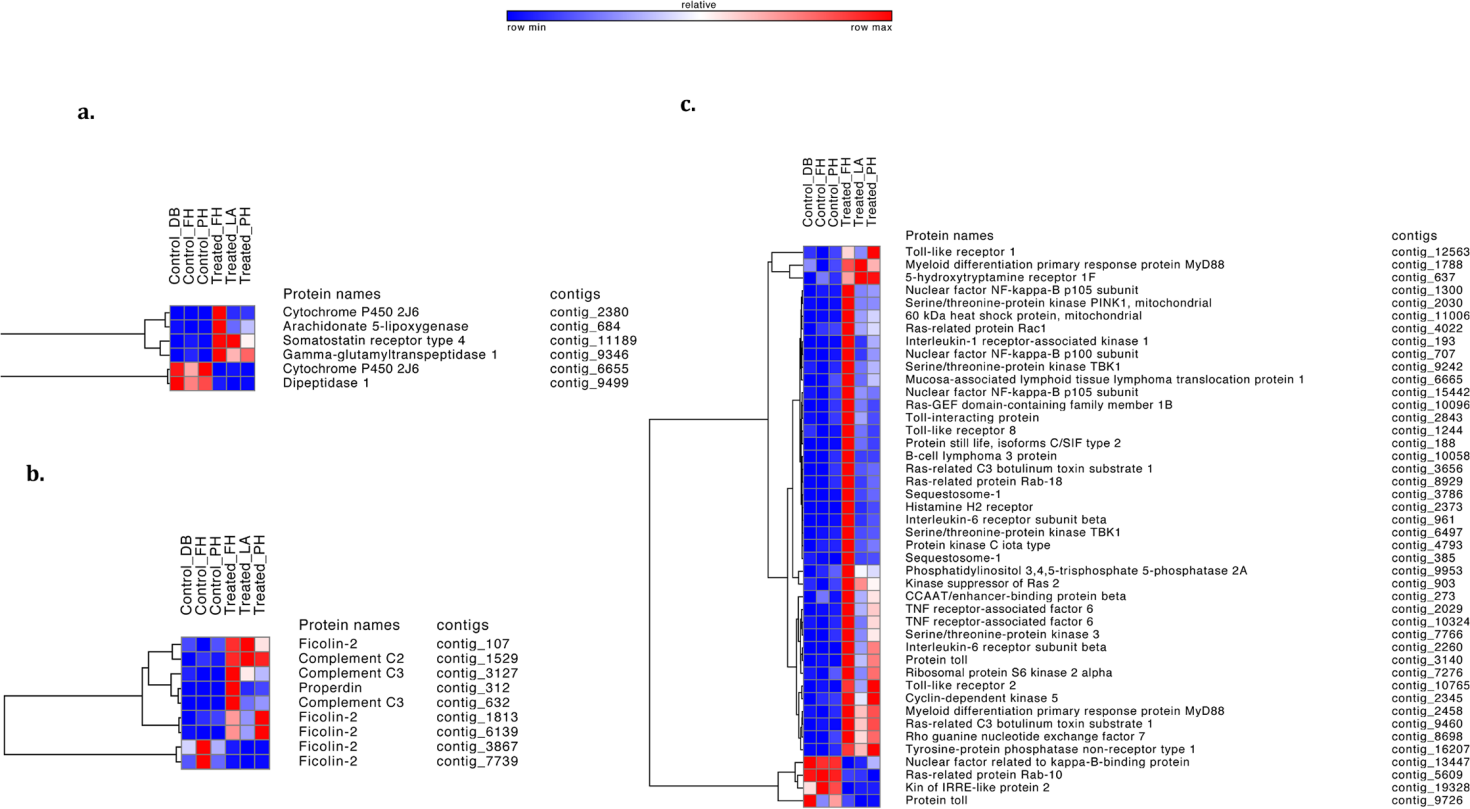
Immune related pathways heatmaps. Heatmaps of immune-related differentially expressed transcripts between control and treated sea stars. Heatmaps are subdivided by related pathway (a) Arachidonic acid metabolism (b) Complement cascade (c) Toll-mediated pathways. Increased expression is shown in red and decreased expression is shown in blue.

**Fig 4:**
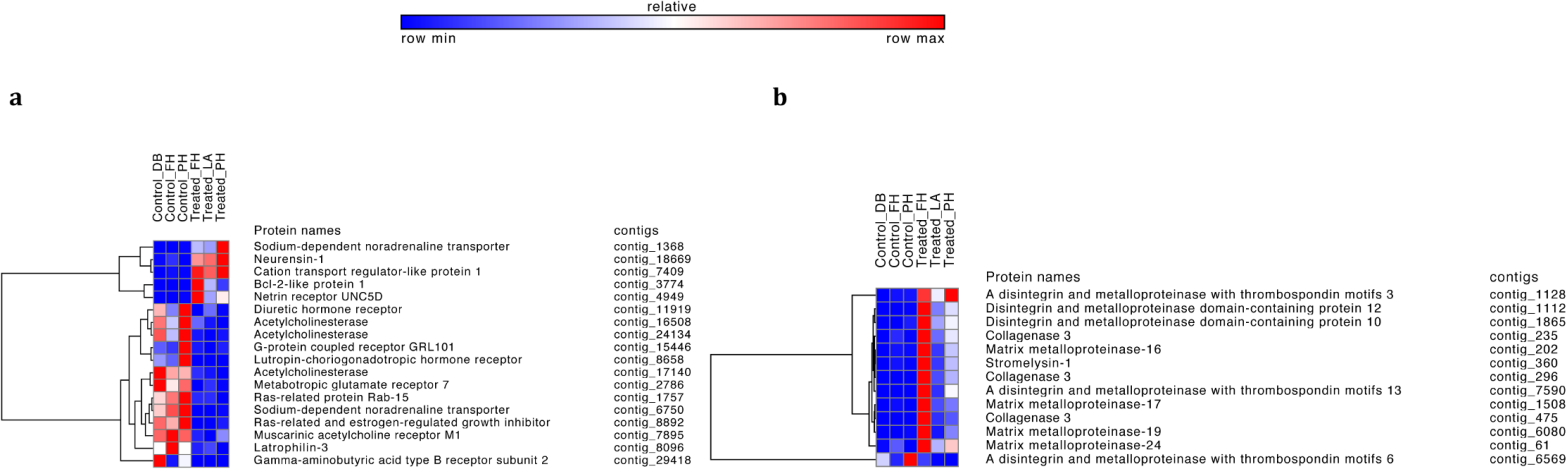
Neural and tissue remodeling pathway heatmaps. Heatmaps of (a) neural and (b) tissue remodeling differentially expressed transcripts between control and treated sea stars.

Heatmaps of contigs that mapped to genes associated with known immune pathways (**Fig 3**) and nervous system growth and organization (**Fig 4**) showed a strong response of the treated sea stars to the viral pathogen. Overall gene expression was higher in treated relative to the control sea stars. Additionally, variation in responses among individual sea stars was also observed. One treated star (Treated_FH) had much higher expression of many of these contigs than the other two treated sea stars. A careful look at the background, collection site, viral load and time course of response did not reveal anything anomalous about this star (Treated_FH). Fold change information and p-values for all contigs can be found in **S1 Table**.

## Discussion

Here we provide the first description of the immune response of an asteroid at the transcriptional level. It is also, as far as we know, the first description of the coelomocyte transcriptomic response of any echinoderm to a natural pathogen [24]. The transcriptome consisted of 29476 consensus sequences, 10513 of which were annotated. A comparison of the *P*. *helianthoides* transcriptome with complete echinoid and asteroid transcriptomes indicates a significant proportion of the transcriptome is represented. As our transcriptome was generated solely from coelomocytes, it is likely that groups of tissue specific transcripts may be missing. Furthermore, due to the long period of exposure to the disease, observed changes in expression may also be, in part, the result of a general stress response as the stars neared their demise. Nevertheless, these data represent a robust transcriptome in a taxonomic class with limited genomic resources, thus providing opportunities to better understand evolution and ecology of asteroid immune systems.

The overarching goal of this effort was to characterize the immune response to the devastating 2013–2014 SSWD outbreak, which has been reported from Alaska to southern California [25]. Clear alteration in gene expression was revealed with over 10% of the contigs differentially expressed. This response is characterized by the increased expression of genes associated with immune pathways such as the Toll pathway, complement cascade, melanization, and arachidonic acid metabolism. In addition to understanding the direct immune response of these sea stars to SSWD, we identified a number of gene expression patterns that likely contribute to observed clinical signs of SSWD. These genes are involved in G-coupled protein processes, WNT signaling, cell adhesion, and neural processes. In several instances, we annotated distinct contigs as the same protein. It is likely these represent different isoforms, different regions of the same gene, or alternatively spliced products. In the remainder of this section we will discuss the primary physiological pathways and gene groups that were influenced by pathogen exposure.

### Toll Pathway

Toll-like receptors (TLRs) have been well characterized in both vertebrates and invertebrates, where they recognize patterns to discriminate between self and non-self, inducing a response to bacterial and viral pathogens [26–29]. A characterization of the sea urchin genome revealed 222 TLRs, some of which likely function in the immune response [9, 30]. While the diversification of TLRs in the sea urchin includes genes that cluster separately from other known TLRs, the high expression in immune cells, the localization to the gut, and the timing of expression reflect involvement in the immune response [31]. Sun et al. (2013) show that sea cucumber TLRs cluster with protostomes and vertebrates and respond to several immune elicitor challenges [32]. Our results strengthen the evidence that echinoderm TLRs play a role in the immune response with the detection of differentially expressed TLRs in stars treated with the SSWD pathogen: TLR1 (contig_12563), TLR2 (contig_10765), and TLR8 (contig_1244). Though the function of these TLRs is likely different from the corresponding TLRs to which they are most similar to at the sequence level, TLR8has been linked to viral recognition in vertebrates [33]. The suite of TLRs differentially expressed in treated sea stars may be a specific response to the denso-virus that is the putative agent of SSWD [14]. Interestingly, Toll-related genes dominated among the immune genes that were differentially expressed in treated vs. control stars, and the annotations are suggestive of several nearly complete Toll-related pathways. This finding suggests that Toll pathways play an important role in the immune response of sea stars to SSWD.

Following recognition, TLRs act through a network of signaling molecules to activate immune and inflammatory cascades [29]. Several of these signaling links appear in the annotations of the differentially expressed genes. For example, one potential pathway following TLR recognition in the treated sea stars is activation of Rac1 (contig_4022), which initiates a pathway to activate NF-kB (contig_707, contig_1300, contig_15442) [34], a transcription factor that triggers multiple immune effectors [35]. This response has been seen in immune-challenged Manila clams [36]. Another Toll pathway gene differentially expressed is Myd88 (contig_2458, contig 1788). Downstream signaling linked to the Myd88 pathway involves a suite of closely interacting genes that are differentially expressed including PINK1 (contig_2030), TRAF6 (contig_2029, contig_10324), Sqstm-1 (contig_385, contig_3786), HSP60 (contig_11086) and MALT1 (contig_6665). The cascade from Myd88 to IRAK1 (contig_193) to TRAF6 is highly conserved and has been found across a wide diversity of metazoans, including mammals, *Drosophila*, shrimp, and urochordates [37, 38]. The pathway even appears to be present and related to immune function in Hydra, a non-bilaterian organism [39, 40]. Previous studies have detected both Myd88 and TRAF6 in sea urchins sea cucumbers [9, 38, 41]. The transcriptomic response to SSWD suggests this signal transduction pathway, as suggested by Hultmark, may play a role in activating the immune response in response upon pathogen challenge in *P*. *helianthoides* [42].

The signaling cascade initiated by TLRs in mammals ultimately leads to the production of interleukins and other inflammatory cytokines through NF-kB [43, 44]. In this experiment we observed evidence of increased interleukin activity in treated sea stars, as there was increased interleukin-6 receptor expression (contig_961, contig_2260). A putative histamine H2 receptor (Hrh2, contig_2373) is also differentially expressed and has been linked to the regulation of IL-10 and IL-12 regulation in mammals [45]. While we identified several putative interleukin-like receptors and binding proteins, only a few contigs were annotated as interleukin proteins (e.g. contig_142—Interleukin 17-like protein, contig_1489—Pro-interleukin-16, and contig_5598—Interleukin 25). The functional roles of IL-6 and H2 receptors may be different in echinoderms, but these results establish a response to the SSWD treatment that spans the breadth of the Toll pathway.

### Complement Cascade

The complement cascade opsonizes pathogens, leading to inactivation and phagocytosis by immune cells and also damages pathogens directly through the formation of the membrane attack complex [35]. Although a previous study into the sea star coelomocyte proteome did not show homologous complement proteins, this study identifies four complement cascade proteins that are differentially expressed. Several contigs have similarity to Ficolin-2 (FCN2; contig_7739, contig_3867, contig_107, contig_1813, contig_6139), Complement C3 (C3; contig_3127, contig_632), Properdin (CFP; contig_312), and Complement C2 (C2; contig_1529). For each component, at least one of the corresponding contigs was expressed at a higher level in treated than control sea stars.

The complement cascade has been well-documented in echinoderms previously and contains C3, which functions in opsonization and phagocytosis, and factor B/C2, which increases C3 production [46, 47]. Here we document the presence of two additional complement cascade proteins, Ficolin-2 and Properdin. Both Ficolin-2 and Properdin induce the complement cascade: Ficolin through association with mannan-binding lectin-associated serine proteases [48], and Properdin by binding to apoptotic cells resulting in complement activation [49]. Not only does this study indicate the complement cascade is an important part of sea star response to wasting disease, but also that echinoderms may have a more complex complement cascade system than previously evidence suggested.

### Melanization

The melanin synthesis cascade in invertebrates is involved in wound healing and immune response by creating chemical/physical barriers and encapsulating pathogens for phagocytosis [50]. One of the contigs with the largest fold change was annotated as a quinone oxidoreductase (CRYZ; contig_9). Similar oxidoreductases (i.e. NQO1) in humans have been described as increasing melanin synthesis by increasing tyrosinase catalytic activity [51]. Various aspects of the melanin synthesis cascade have been documented in a number of Echinoderms [52]. Previous experiments using sheep erythrocytes as an immune challenge in the sea cucumber *Holothuria polii* resulted in both clearance of the antigen and the production of ‘brown masses’, which were positive for Schmorl’s, Lillie’s, and Hueck’s reactions, indicating the presence of melanin [52]. Furthermore, prophenoloxidase, an important part of the melanin synthesis cascade, has been detected in echinoderms, though at lower levels than those observed in other invertebrates [2]. Our results serve as the first gene based evidence of melanin synthesis in an echinoderm and suggests melanin production may play an important role in healing of lesions of infected sea stars.

### Arachidonic Acid Metabolism

While not well characterized in echinoderms, arachidonic acid metabolism is involved in many immune-specialized cells in humans and other vertebrates and can induce phagocytosis, inflammation, pain, and chemotaxis [53–55]. Arachidonic acid metabolism has recently been associated with the immune response of the staghorn coral, *Acropora cervicornis* to white band disease, suggesting a role for this pathway in immunity in metazoans diverging earlier on the phylogenetic tree [56]. We identified six differentially expressed transcripts associated with arachidonic acid metabolism. Arachidonate 5-lipoxygenase (contig_684), which displayed increased expression in treated sea stars plays an integral part in the arachidonic acid pathway, metabolizing arachidonic acid into 5-HPETE which is later converted to leukotrienes, which modulate leukocyte activity [55]. Two other differentially expressed genes, Dipeptidase 1 (contig_9499) and gamma-glutamyl transpeptidase 1 (contig_9346) are both involved in biosynthesis of leukotrienes [57]. Two contigs identified as cytochrome P450 2J6 (contig_2380 and contig_6655) were differentially expressed in treated vs control stars. Cytochrome P450 2J6 catalyzes the NADPH-dependent oxidation of arachidonic acid [58].

### Nervous system activity

Observable signs of SSWD include a deflated appearance, twisting arms, lesions and, in advanced cases, arm autotomy and death [12]. In our experiment all treated sea stars exhibited the signs associated with SSWD. The nervous system is the primary controller of adhesion and connective tissues, including the mutable collagenous tissues (MCT), which help maintain sea star structure [59]. Therefore the signs of SSWD suggest a role for the nervous system and connective tissues in the disease pathology.

Numerous differentially expressed genes identified here suggest a disruption of neural function, possibly having downstream effects on connective tissue function. Norepinephrine transporters (contig_1368, contig_6750), which are responsible for the uptake of the stress hormone norepinephrine into synaptic terminal were differentially expressed. BCL-2-like protein 1 (BCLx, contig_3774), which is involved with apoptosis [60, 61] and regulation of synaptic activity [62], was expressed at a higher level in treated sea stars. There is also evidence for neurogenesis, as nerve guidance and growth protein transcripts such as Blocks Notch protein (Botch, contig_7409) [63], neurensin (contig_18669) [64], and netrin receptor UNC5D (contig_4949) [65] were all highly expressed in treated sea stars.

G-protein coupled receptors (GPCRs) were exclusively expressed at lower levels in treated sea stars and showed suppression of signaling cascades compared to control sea stars. One such gene is gamma-aminobutyric acid B receptor 2 (GABA_B_R2, contig_29418), a neuroinhibitor that helps fine-tune neural function [66]. Other GPCRs observed and involved with neural processes included calcium-independent alpha-latrotoxin receptor 3 (contig_8096), and GRL101 (contig_15446). These among others were found in the *S*. *purpuratus* (sea urchin) and *Saccoglossus kowalevskii* (acorn worm) genomes and linked to neural processes [67, 68]. Additionally, estrogen-regulated growth inhibitor (contig_8892) and Ras-related protein Rab-15 (contig_1757), both of which bind GTP for activation of G protein, were identified and expressed at lower levels in treated sea stars. GTP activates G proteins of subsequent signaling pathways within the cell [69]. Without the activation of these processes, subsequent signaling cascades cannot occur.

The neurotransmitter acetylcholine is known to be a stiffening modulator of MCT in urchins [70, 71] Acetylcholine’s degrading enzyme, acetylcholinesterase (contig_16508, contig_17140, contig_24134) and GPCR muscarinic acetylcholine receptor M (contig_7895) are both expressed at lower levels in the treated sea stars. Given the dramatic morphological alterations associated with SSWD (i.e. loss of arms) it is not surprising that that we see a signature of acetylcholine signaling changes. Additional gene expression patterns that correlated with the signs of the disease include differential expression of several GPCRs known to activate adenylate cyclase activity [Luteinizing hormone receptor (contig_8658), Metabotropic glutamate receptor 7 (contig_2786), and Diuretic hormone receptor (contig_11919)]. Adenylate cyclase has been shown to cause muscle relaxation in sea stars by possibly mediating the nitric oxide signaling pathway [72]. However, it is not clear whether the clinical signs of SSWD, and underlying molecular mechanisms, are associated with a host response and/or controlled by the causative agent.

### Tissue remodeling

Groups of genes involved in tissue remodeling and wound healing were also differentially expressed in control and treated stars. These genes are especially exciting, as they suggest possible underlying mechanisms of SSWD signs such as the “melted” appearance and loss of arms. Extra-cellular matrix (ECM) connective tissue remodeling occurs through matrix metalloproteinases (MMPs) and disintegrin proteolysis [73]. MMPs, such as collagenase and stromelysin, degrade components of the ECM and are essential in tissue regeneration and remodeling following gut evisceration in the sea cucumber *Holothuria glaberrima*, indicating a key role in mutable connective tissue (MCT) changes that may lead to some of the signs exhibited in SSWD [70, 74]. In the treated stars, both collagenase-3 (contig_235, contig_296, contig_475) and stromelysin (contig_360) were expressed at a higher level. Additionally, 9 other proteases change expression with treatment, disintegrins ADAM-10 (contig_1865) and ADAM12 (contig_1112), the ADAMTS proteins ADAM-TS3, (contig_1128), ADAM-TS6 (contig_6569), and ADAM-TS13 (contig_7590), and the membrane-associated metalloproteinases MMP-3 (contig_360), MMP-13 (contig_235, contig_296, contig_475), MMP-16 (contig_202), MMP-17 (contig_1508), MMP19 (contig_6080), and MMP24 (contig_61). Increased (differential) expression of the disintegrins and MMPs was observed in the treated sea stars except for ADAM-TS6, indicating a massive MCT response. Our finding of tissue remodeling being impacted by SSWD is consistent with Gudenkauf and Hewson’s recent metatranscriptomic analysis [75]. A significant amount of additional research is needed to understand the bizarre and dramatic pathogenesis of SSWD and to pinpoint the degree to which the host and the pathogen are each responsible for controlling the observed “melting” phenotype. Whether this phenotype results from physiological changes induced by the causative agent or represents an attempt at a host response is not known.

## Conclusion

The widespread impacts of SSWD warrant a greater understanding of the invertebrate and echinoderm immune system. Though several questions remain regarding the epidemiology and molecular mechanisms related to this devastating disease, our study of the *P*. *helianthoides* transcriptome provides the first characterization of the transcriptional immune response of an asteroid. Moreover, it is the first to use a natural pathogen in the study of the echinoderm transcriptional immune response, thus bolstering the existing immune resources for marine invertebrates. The work presented here provides unambiguous evidence for a robust host response in treated individuals and provides new clues to the active immune components in the asteroid immune response. Our comparative study of *P*. *helianthoides* provides insights into the mechanisms underlying the “wasting” phenotype, such as changes in expression of genes involved with adhesion and disruption to neural-related functions. Future research could further explore the connection between these physiological responses, SSWD, and the sea star immune system using sea stars sampled from a larger area. This project builds a strong foundation for additional immune-focused studies in *P*. *helianthoides* and other asteroids. The data offer an important genomic resource for future assessments of echinoid health. This study offers a detailed account of the sea star response during the initial stages of the 2013–2014 SSWD epidemic in Washington state and provides new candidate immune pathways for evaluating impacts of natural selection on the population over the course of this epidemic.

## Supporting Information

S1 Table Summary of Gene Expression Data. (TAB)
